# Supplementary material for: YAP1 and TAZ negatively control bone angiogenesis by limiting hypoxia-inducible factor signaling in endothelial cells
Source: eLife. 2020 Jan 20;9:e50770. doi: 10.7554/eLife.50770 (PMC6970532; doi:10.7554/eLife.50770)
Supplement: Supplementary file 1. [file elife-50770-supp1.docx]

| **Key Resources Table** | | | | |
| --- | --- | --- | --- | --- |
| **Reagent type (species) or resource** | **Designation** | **Source or reference** | **Identifiers** | **Additional information** |
| strain, strain background (*Mus musculus*, C57BL/6JRj) | WT | Janvier Labs |  |  |
| genetic reagent (*Mus musculus*) | *Cdh5-mTnG* | this paper |  | endothelial cell reporter mice; (express membrane-anchored tdTomato and nuclear H2B-GFP);  see Methods for details; line available from the Adams lab upon request |
| genetic reagent (*Mus musculus*) | *Cdh5-CreERT2* | Wang et al., 2010 |  |  |
| genetic reagent (*Mus musculus*) | *Yap1^flox^* | Reginensi et al., 2013 |  |  |
| genetic reagent (*Mus musculus*) | *Taz^flox^* | Reginensi et al., 2013 |  |  |
| genetic reagent (*Mus musculus*) | Rosa26-*Yap1^S112A^* | this paper |  | Inducible overexpression of stabilized Yap1; see Methods for details; line available from the Adams lab upon request |
| genetic reagent (*Mus musculus*) | *Lats2^flox^* | Lu et al., 2010 |  |  |
| genetic reagent (*Mus musculus*) | *Rosa26-HIF1A^dPA^* | The Jackson Laboratory | JAX: 009673 |  |
| genetic reagent (*Mus musculus*) | *Hif1a^flox^* | The Jackson Laboratory | JAX: 007561 |  |
| cell line (*Homo sapiens*) | Human Umbilical Vein Endothelial Cells (HUVEC) | ThermoFisher | Cat# C0035C |  |
| transfected construct (*Homo sapiens*) | Negative control siRNA | Ambion | Cat# 4390844 |  |
| transfected construct (*Homo sapiens*) | *YAP1* siRNA | Ambion | Cat# 4392420  (ID:s20366) |  |
| transfected construct (*Homo sapiens*) | *YAP1* siRNA | Ambion | Cat# 4392420  (ID:s20367) |  |
| transfected construct (*Homo sapiens*) | *WWTR1* siRNA | Ambion | Cat# 4392420  (ID:s42789) |  |
| transfected construct (*Homo sapiens*) | *WWTR1* siRNA | Ambion | Cat# 4392420  (ID:s42787) |  |
| transfected construct (*Homo sapiens*) | *HIF1A* siRNA | Ambion | Cat# 4392420  (ID:s6539) |  |
| antibody | Rat monoclonal anti-Endomucin (V.7C7) | Santa Cruz | Cat# sc-65495 RRID:AB_2100037 | IF (1:100) |
| antibody | Goat polyclonal anti-CD31 | R&D | Cat# AF3628  RRID:AB_2161028 | IF (1:100) |
| antibody | Rabbit monoclonal anti-Yap (DBH1X) | Cell signaling | Cat# 14074 RRID:AB_2650491 | IF (1:100)  WB (1:1000) |
| antibody | Rabbit polyclonal anti-Wwtr1 | Sigma-Aldrich | Cat# HPA007415 RRID:AB_1080602 | IF (1:100)  WB (1:1000) |
| antibody | Rabbit polyclonal anti-Lats1/2 | Bethyl | Cat#A300-479A RRID:AB_2133375 | IF (1:100) |
| antibody | Rabbit monoclonal anti-Yap(ser127) | Cell Signaling | Cat# 13008 RRID:AB_2650553 | IF (1:50) |
| antibody | Rabbit monoclonal anti-Yap1/Taz (D24E4) | Cell Signaling | Cat# 8418 RRID:AB_10950494 | IF (1:100)  WB (1:1000) |
| antibody | Goat polyclonal anti-CD31 (APC-conjugated) | R&D | Cat# FAB3628A RRID:AB_10971931 | IF (1:100) |
| antibody | Mouse monoclonal anti-alpha-smooth muscle actin (Cy3 - conjugated) | Sigma-Aldrich | Cat#C6198 RRID:AB_476856 | IF (1:200) |
| antibody | Rabbit polyclonal anti-Hif-2α | Novus Biologicals | Cat# NB100-122 RRID:AB_10002593 | IF (1:100) |
| antibody | Rabbit polyclonal anti-HIF-1alpha | Santa Cruz | Cat# sc-10790 RRID:AB_2116990 | IF (1:100) |
| antibody | Rabbit polyclonal anti-HIF-1alpha | Thermo Scientific | Cat#PA1-16601 RRID:AB_2117128 | IF (1:100)  WB(1:250)  IP |
| antibody | Goat polyclonal anti-VEGFR3 | R&D | Cat# AF743 RRID:AB_355563 | IF (1:100) |
| antibody | Rabbit polyclonal anti-OSX (A-13) | Santa Cruz | Cat# sc-22536-R  RRID:AB_831618 | IF (1:200) |
| antibody | Rabbit polyclonal anti-OSX | Abcam | Cat#ab22552 RRID:AB_2194492 | IF (1:500) |
| antibody | Rabbit monoclonal anti-Erg | Abcam | Cat#ab110639 RRID:AB_10864794 | IF (1:100) |
| antibody | Rabbit monoclonal anti-cleaved Caspase-3(ASP175) | Cell Signaling | Cat# 9664RRID:AB_2070042 | IF (1:100) |
| antibody | Goat polyclonal anti-Osteopontin | R&D | Cat# AF808 RRID:AB_2194992 | IF (1:200) |
| antibody | Rabbit polyclonal anti-Osteocalcin | Lifespan Bioscience | Cat# LS-C17044 RRID:AB_798874 | IF (1:500) |
| antibody | Rabbit polyclonal anti-Collagen type 1 | Millipore | Cat# AB765P RRID:AB_11212199 | IF (1:100) |
| antibody | Rabbit polyclonal anti-Runx2 (M-70) | Santa Cruz | Cat# sc-10758 RRID:AB_2184247 | IF (1:50) |
| antibody | Isolectin-B4 (IB4) | Vector | Cat#B-1205  RRID:AB_2314661 | IF (1:100) |
| antibody | Rabbit monoclonal anti-ATP6VIB1&2 | Abcam | Cat#ab200839 | IF (1:100) |
| antibody | Anti rat Alexa Fluor 488 | ThermoFisher Scientific | Cat#A21208 RRID:AB_141709 | IF (1:200) |
| antibody | Anti rat Alexa Fluor 594 | ThermoFisher Scientific | Cat#A21209 RRID:AB_2535795 | IF (1:200) |
| antibody | Anti goat Alexa Fluor 546 | ThermoFisher Scientific | Cat#A11056 RRID:AB_142628 | IF (1:200) |
| antibody | Anti goat Alexa Fluor 647 | ThermoFisher Scientific | Cat#A21447 RRID:AB_141844 | IF (1:200) |
| antibody | Anti rabbit Alexa Fluor 647 | ThermoFisher Scientific | Cat#A31573 RRID:AB_2536183 | IF (1:200) |
| antibody | Anti rabbit Alexa Fluor 488 | ThermoFisher Scientific | Cat#A21206 RRID:AB_141708 | IF (1:200) |
| antibody | Streptavidin Alexa Fluor-488 | Invitrogen | Cat# S11223 | IF (1:100) |
| antibody | Streptavidin Alexa Fluor-647 | Invitrogen | Cat# S32357 | IF (1:100) |
| sequence-based reagent  (*Homo sapiens*) | Eukaryotic 18S rRNA Endogenous Control (VIC™/MGB probe, primer limited) | ThermoFisher | Cat#4319413E | TaqMan probe Hs99999905_m1 |
| sequence-based reagent  (*Mus musculus*) | *Vegfa* | ThermoFisher | Cat# 4331182 | (Mm00437306_m1) |
| sequence-based reagent  (*Mus musculus*) | *Angptl4* | ThermoFisher | Cat# 4331182 | (Mm00480431_m1) |
| sequence-based reagent  (*Mus musculus*) | *Ctgf* | ThermoFisher | Cat# 4331182 | (Mm01192932_g1) |
| sequence-based reagent  (*Mus musculus*) | *Cyr61* | ThermoFisher | Cat# 4331182 | (Mm00487499_g1) |
| sequence-based reagent  (*Mus musculus*) | *Lats2* | ThermoFisher | Cat# 4331182 | \| (Mm00497217_m1 ) \|  \| \| --- \| --- \| |
| sequence-based reagent  (*Homo sapiens*) | *VEGFA* | ThermoFisher | Cat# 4331182 | (Hs00900055_m1) |
| sequence-based reagent  (*Homo sapiens*) | *CTGF* | ThermoFisher | Cat# 4331182 | (Hs01026927_g1) |
| sequence-based reagent  (*Homo sapiens*) | *CYR61* | ThermoFisher | Cat# 4331182 | (Hs00998500_g1) |
| sequence-based reagent  (*Homo sapiens*) | *VEGFA* | ThermoFisher | Cat# 4331182 | (Hs00900055_m1) |
| sequence-based reagent  (*Homo sapiens*) | *ANGPTL4* | ThermoFisher | Cat# 4331182 | (Hs01101127_m1) |
| sequence-based reagent  (*Homo sapiens*) | *IGFBP2* | ThermoFisher | Cat# 4331182 | (Hs01040719_m1) |
| sequence-based reagent  (*Homo sapiens*) | *XBP1* | ThermoFisher | Cat# 4331182 | (Hs00231936_m1) |
| sequence-based reagent  (*Homo sapiens*) | *YAP1* | ThermoFisher | Cat# 4331182 | (Hs00902712_g1) |
| sequence-based reagent  (*Homo sapiens*) | *WWTR1 (TAZ)* | ThermoFisher | Cat# 4331182 | (Hs00210007_m1) |
| sequence-based reagent  (*Homo sapiens*) | *HIF1A* | ThermoFisher | Cat# 4331182 | (Hs00153153_m1) |
| sequence-based reagent  (*Mus musculus*) | *Yap1_F* | this paper | PCR Primer | \| TGAGATCCCTGATGATGTACCAC \|  \| \| --- \| --- \|   see Methods |
| sequence-based reagent  (*Mus musculus*) | *Yap1_R* | this paper | PCR Primer | TGTTGTTGTCTGATCGTTGTGAT  see Methods |
| sequence-based reagent  (*Mus musculus*) | *Wwtr1_F* | this paper | PCR Primer | AGTCCTATVACGTGACCGACGGA  see Methods |
| sequence-based reagent  (*Mus musculus*) | *Wwtr1_R* | this paper | PCR Primer | \| GGTCTTGCCATGTGGTGATTTT \|  \| \| --- \| --- \|   see Methods |
| sequence-based reagent  (*Homo sapiens*) | *VEGFA* (-947)_F | this paper | PCR Primer | GCCAGACTCCACAGTGCATA  see Methods |
| sequence-based reagent  (*Homo sapiens*) | *VEGFA* (-947)_R | this paper | PCR Primer | CTGAGAACGGGAAGCTGTGT  see Methods |
| commercial assay or kit | Hypoxiprobe Plus kit-FITC | Hypoxiprobe | Cat# HP2 |  |
| commercial assay or kit | Click-iT EdU Alexa-647 imaging kit | Invitrogen | Cat# C10340 |  |
| commercial assay or kit | RNeasy Plus Mini Kit | QIAGEN | Cat# 74134 |  |
| commercial assay or kit | iScript cDNA Synthesis kit | BIO-RAD | Cat# 1708890 |  |
| commercial assay or kit | Lipofectamine RNAiMAX | Invitrogen | Cat# 13778075 |  |
| commercial assay or kit | TaqMan Gene Expression Master Mix | Thermoscientific | Cat#4369016 |  |
| commercial assay or kit | PowerUp SYBR Green Mater Mix | Thermoscientific | Cat#A25742 |  |
| commercial assay or kit | Quantification-RNA (Bioanalyzer) | Thermoscientific | Cat#Q32851 |  |
| commercial assay or kit | MiSeq Reagent Kit v3 | Illumina | Cat#MS-102-3001 |  |
| chemical compound, drug | MG-132 proteasome inhibitor | Millipore | Cat# 474790 |  |
| chemical compound, drug | Tamoxifen | Sigma | Cat#T5648 |  |
| chemical compound, drug | 4’,6’-diamidino-2-phenylindole (DAPI) | Sigma | Cat# D9542 |  |
| chemical compound, drug | Sucrose | Sigma | Cat# S0389 |  |
| chemical compound, drug | cOmplete ULTRA Tablets Protease Inhibitor Cocktail | Roche | Cat# 05892970001 |  |
| chemical compound, drug | phosphatase inhibitor cocktail set V | EMD Millipore | Cat# 524629 |  |
| chemical compound, drug | Gelatine | Sigma | Cat# G1890 |  |
| chemical compound, drug | Polyvinylpyrrolidone | Sigma | Cat# P5288 |  |
| chemical compound, drug | Fatty-acids free BSA | Sigma | Cat# A9205 |  |
| chemical compound, drug | Trypsin-EDTA solution | Sigma | Cat# T3924 |  |
| chemical compound, drug | Paraformaldehyde | Sigma | Cat# P6148 |  |
| chemical compound, drug | Fluoromount-G | Southern Biotech | Cat# 0100-01 |  |
| chemical compound, drug | ECL™ Prime Western Blotting Detection Reagent | GE-Healthcare | Cat# RPN2236 |  |
| chemical compound, drug | Dimethyl sulfoxide | Sigma | Cat# D8418 |  |
| chemical compound, drug | EBM-2 endothelial cells medium | Lonza | Cat# CC-3156 |  |
| chemical compound, drug | EGM-2 Single Quots | Lonza | Cat# CC-4176 |  |
| software, algorithm | Volocity (v6.3) | Perkin Elmer | RRID:SCR_002668 |  |
| software, algorithm | GraphPad Prism7 | GraphPad Software | RRID:SCR_002798 |  |
| software, algorithm | FlowJo (v10.3) | BD Life Sciences | RRID:SCR_008520 |  |
| software, algorithm | Illustrator (vCC2018) | Adobe | RRID:SCR_010279 |  |
